# Could “the bloomy rind sign” be associated with meningeal dissemination of lung adenocarcinoma and varicella zoster virus infection? A case report and literature review

**DOI:** 10.1186/s12883-026-05265-x

**Published:** 2026-08-12

**Authors:** Ryotaro Hida, Saro Kobayashi, Keisuke Suzuki

**Affiliations:** 1 Department of Neurology, Dokkyo Medical University, Kitakobayashi 880, Mibu, Shimotsuga, Tochigi, Japan; 2 Stroke Center, Dokkyo Medical University Hospital, Mibu, Japan

**Keywords:** Bloomy rind sign, Lung adenocarcinoma, Meningeal dissemination, Varicella-zoster virus, Magnetic resonance imaging, Literature review

## Abstract

**Background:**

The bloomy rind sign, also described as a band-like lesion, is a characteristic imaging finding observed in a subset of patients with meningeal dissemination from lung adenocarcinoma (LA). However, its mechanism and clinical significance remain unclear.

**Methods:**

We report a case in which LA with varicella-zoster virus (VZV) meningitis presented with a bloomy rind sign. A literature review was conducted to analyze the clinical and radiological characteristics of the reported cases.

**Results:**

A 48-year-old man with stage IVB LA developed VZV meningitis and exhibited a characteristic lesion extending from the ventral pons to the cerebellar peduncles, showing hyperintensity on diffusion-weighted imaging (DWI) and fluid-attenuated inversion recovery, reduced apparent diffusion coefficient values, and no contrast enhancement. A review of 27 reported cases revealed highly consistent imaging findings, with DWI hyperintensity in all evaluated patients (26/26, 100%) and T2-weighted hyperintensity in all patients (27/27, 100%), whereas contrast enhancement was rare (1/26, 3.8%). The clinical outcomes were poor, with a mortality rate of 63.2%. Cerebrospinal fluid cytology was negative in 36.0% of the patients. The only nonneoplastic case presenting with the bloomy rind sign was associated with VZV meningitis, and VZV testing was performed in only 8.3% of LA-associated cases. The present patient was VZV positive.

**Conclusion:**

The bloomy rind sign has consistent MRI features and may be a useful imaging marker for the early diagnosis of meningeal dissemination from LA. The present case raises the possibility that VZV infection may contribute to the development of the bloomy rind sign; however, this hypothesis is based on a single case and requires further validation.

## Introduction

A characteristic imaging finding observed in patients with meningeal dissemination from lung adenocarcinoma (LA) is a hyperintense lesion extending from the anterior pons to the cerebellar peduncles on diffusion-weighted imaging (DWI) and fluid-attenuated inversion recovery (FLAIR) sequences [1]. These imaging findings are referred to as the bloomy rind sign or band-like lesion [2]. Although several cases have been reported to date, this imaging feature is observed only in a subset of patients with LA complicated by meningeal dissemination. Consequently, many clinical questions remain unsolved, including the mechanism underlying this phenomenon and why it occurs only in a subset of patients with meningeal dissemination. We report the case of a patient with stage IVB LA who developed varicella-zoster virus (VZV) meningitis and exhibited a bloomy rind sign (band-like lesion). In addition, through a review of the literature, we explore the mechanism of this lesion in the context of this case.

## Methods

A literature search was performed in PubMed using the terms “bloomy rind sign,” “band-like lesion,” “band-like hyperintensity,” “lung adenocarcinoma,” and “leptomeningeal metastasis”. The reference lists of relevant articles were also reviewed to identify additional reports. Cases with sufficient individual clinical information, including imaging findings, cerebrospinal fluid cytology, treatment, and clinical course, were included in the pooled analysis. For the case series, only individually described cases with sufficient clinical information were included.

### Case presentation

**Fig. 1:**
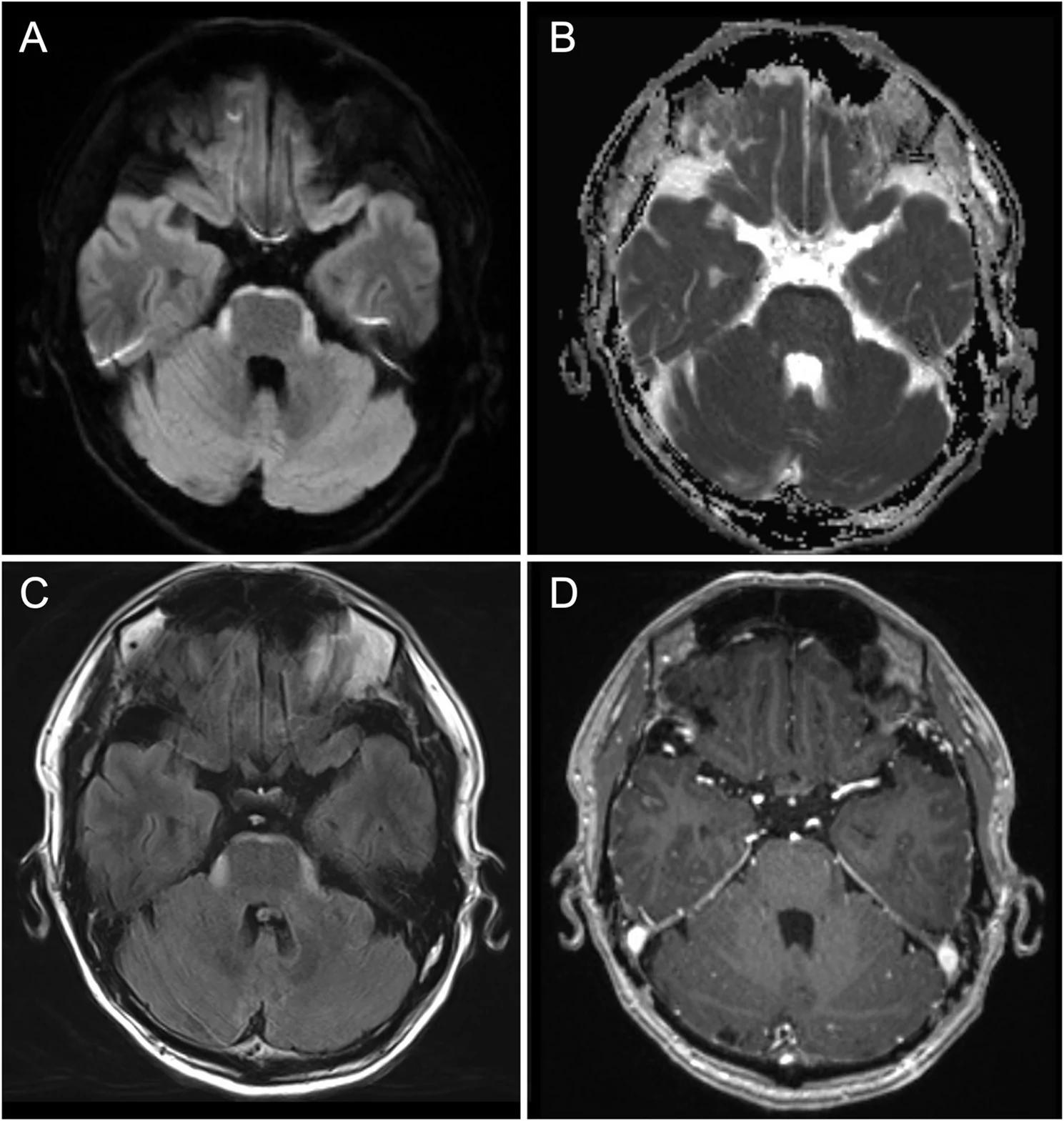
Brain MRI of the patient. **A** Brain MRI reveals a high-intensity lesion around the ventral surface of the pons extending to the middle cerebellar peduncles on DWI. **B** The corresponding area shows reduced ADC values. **C** The lesion is hyperintense on FLAIR. **D** No contrast enhancement is observed in the brainstem lesion on postcontrast T1-weighted images

A 48-year-old man with a few-week history of headaches and nausea presented at the outpatient clinic. He had a two-year history of stage IVB LA with epidermal growth factor receptor (EGFR) mutations. He had been treated with osimertinib, an EGFR tyrosine kinase inhibitor, in combination with carboplatin and pembrolizumab. During the course of treatment, brain magnetic resonance imaging (MRI) revealed brain parenchymal metastasis; thus, osimertinib was changed to erlotinib, and the patient was monitored. However, his headaches and nausea gradually worsened. On admission, neurological evaluation revealed neck stiffness, disturbance of consciousness, and anisocoria. There were no findings suggestive of facial nerve involvement, including facial palsy, sensorineural hearing loss, tinnitus, vertigo, nystagmus, or herpetic vesicular rash around the ear. He had a rash with crusting in the left L3 dermatome of the thigh. Blood tests revealed elevated white blood cell counts and C-reactive protein, carcinoembryonic antigen, and sialyl Lewis X-i antigen levels. Cerebrospinal fluid (CSF) examination revealed pleocytosis (400 polymorphonuclear cells/µL and 85 mononuclear cells/µL), elevated protein levels (1,274.6 mg/dL), and a positive PCR test for VZV. No malignant cells were detected in either of the two CSF cytology examinations. CSF bacterial and mycobacterial cultures were negative. The CSF sIL-2R and ACE levels were 2,293.4 U/mL and 2.2 U/L, respectively. Brain MRI revealed a high-intensity lesion along the ventral surface of the pons extending to the middle cerebellar peduncles on DWI and FLAIR images, and the apparent diffusion coefficient (ADC) value in the corresponding region was reduced. However, there was no contrast enhancement of this brainstem lesion on postcontrast T1-weighted images (Fig. 1). After the administration of acyclovir, his level of consciousness temporarily improved but subsequently deteriorated along with his clinical condition. Follow-up brain MRI performed 12 days after the initial examination revealed no change in the brainstem lesion and no contrast enhancement. Subsequent MRI performed 49 days after the initial examination again demonstrated an unchanged brainstem lesion without contrast enhancement but revealed newly developed hydrocephalus. A third MRI obtained 76 days after the initial examination revealed a persistent rind sign with no contrast enhancement and further progression of hydrocephalus. The patient was subsequently transferred to another hospital for supportive care.

## Discussion

Our patient’s characteristic MRI findings revealed a lesion extending from the ventral surface of the pons to the cerebellar peduncles. This lesion showed hyperintensity on FLAIR and DWI and a low ADC value. On the basis of the patient’s clinical background, this lesion was considered to reflect meningeal dissemination of LA, but no contrast enhancement was observed. This finding, referred to as the bloomy rind sign or a band-like lesion, has been reported as a characteristic imaging finding of meningeal dissemination of LA, although its mechanism remains unclear. Previous reports suggest that tumor cells infiltrate the brain surface, leading to microinfarcts and inflammatory changes [1, 3]. However, the lesion does not correspond to a vascular territory, and several reported patients showed no leptomeningeal enhancement on postcontrast T1-weighted images.

In an autopsy case of a patient with LA with meningeal dissemination, destruction of the vascular structure because of infiltration of tumor cells was observed in areas where the surface of the brain was enhanced. In contrast, tumor cells have been reported to accumulate in the perivascular space in areas without contrast enhancement [4], suggesting that in patients with the bloomy rind sign, similar perivascular tumor infiltration in the ventral brainstem may result in hyperintense lesions on DWI and FLAIR without contrast enhancement. Furthermore, CSF circulation may be involved in the mechanism underlying lesions confined to the pontine surface. The prepontine cistern, a subarachnoid space located ventral to the pons, is surrounded by a thin membrane known as the Liliequist membrane; anatomically, this structure may be prone to CSF stasis [5].

The mechanism underlying the nonenhancing nature of the bloomy rind sign remains unclear. Tan et al. [6] proposed that the bloomy rind sign may represent a treatment-related imaging finding because most reported cases occurred in patients who had received targeted therapies, including tyrosine kinase inhibitors and antiangiogenic agents such as bevacizumab. Since leptomeningeal metastases usually demonstrate contrast enhancement owing to increased vascularity and disruption of the blood–brain barrier, inhibition of tumor neovascularization by these therapies may result in nonenhancing leptomeningeal metastases. However, Cheng et al. [7] reported a patient in whom the bloomy rind sign was the initial manifestation leading to the diagnosis of lung adenocarcinoma before the initiation of systemic therapy. Therefore, treatment-related vascular changes alone are unlikely to explain all cases, and the underlying mechanism of the bloomy rind sign remains to be elucidated.

This is the first reported case of a patient who developed a bloomy rind sign with the co-occurrence of meningeal dissemination of LA and VZV meningitis. We summarized the characteristics of previously reported cases of the bloomy rind sign (Table 1) [1–3, 6–23]. In 25 of 27 patients (92.6%), the primary disease was LA, indicating that these imaging findings are highly specific to meningeal dissemination of LA. Similarly, Mitsuya et al. [2] conducted a retrospective single-center study of 142 patients with solid cancer who developed meningeal dissemination and reported that the bloomy rind sign was observed in 11 patients, all of whom had LA. Furthermore, in 18.5% of patients (5/27), brain lesions were detected prior to the diagnosis of LA, following the onset of meningitis symptoms such as fever and headache [11, 13, 15, 21, 23]. Hyperintensity on DWI was observed in all of the evaluated patients (26/26, 100%), and T2-weighted imaging demonstrated hyperintensity in all of the patients (27/27, 100%). Notably, contrast enhancement was observed in only one patient (1/26, 3.8%). As 9 of 25 patients (36.0%) whose CSF cytology data were available tested negative, despite CSF cytology being the gold standard for diagnosing meningeal dissemination, the bloomy rind sign may represent a useful imaging marker for the early detection of meningeal dissemination from LA. Clinical outcomes were variable; however, among patients with available outcome data, approximately two-thirds of the reported patients (63.2% (12/19)) died, suggesting that these imaging findings may be associated with poor prognosis. Among the reported cases, the only nonneoplastic case in which the bloomy rind sign was present was associated with VZV meningitis [15]. Among the 24 patients with LA and the bloomy rind sign, only two (8.3%), including the current patient, underwent VZV testing of the CSF. Given that the current patient was diagnosed with meningeal dissemination of LA complicated by VZV meningitis, it is possible that the combination of meningeal dissemination of LA and VZV meningitis may increase the likelihood of developing the bloomy rind sign. Furthermore, whether VZV was detected in the CSF of other patients with LA-associated bloomy rind signs on MRI is unclear, suggesting that the potential contribution of VZV infection may not have been recognized. VZV is known to cause a wide spectrum of central nervous system complications, including meningitis, encephalitis, and vasculopathy [24].

**Table 1:** Clinical and radiological characteristics of reported cases of bloomy rind signs or band-like lesions

Authors	Age (yr)	Sex	Primary disease	EGFR mutation	Symptoms	DWI	ADC	T2WI/FLAIR	Gd-T1WI	CSF cytology	CSF VZV	Prognosis
Crombe et al. [1]	56	N/A	LA	positive	N/A	high	low	hyperintense	enhanced	positive	N/A	died
Mitsuya et al. [2]	N/A	F	LA	N/A	headache, nausea	high	N/A	hyperintense	not enhanced	positive	N/A	alive
Khil et al.[3]	75	M	LA	N/A	headache, dizziness	high	N/A	hyperintense	not enhanced	positive	N/A	died
	47	F	LA	N/A	N/A	high	low	hyperintense	unclear	N/A	N/A	alive
Cheng et al. [7]	39	M	LA	positive	dizziness, tinnitus, hearing impairment, seizure, unsteady gait	high	low	hyperintense	not enhanced	positive	N/A	died
	N/A	N/A	LA	positive	dizziness, unsteady gait, seizures	high	low	hyperintense	not enhanced	N/A	N/A	lost to follow up
	N/A	N/A	LA	positive	slurred speech, swallowing difficulties, general weakness	high	low	hyperintense	not enhanced	negative	N/A	died
	N/A	N/A	LA	positive	dizziness, unsteady gait, generalized weakness	high	low	hyperintense	not enhanced	N/A	N/A	alive
Maeda et al. [8]	55	M	LA	positive	dizziness, vomiting	high	low	hyperintense	not enhanced	positive	N/A	N/A
	77	M	LA	N/A	N/A	high	low	hyperintense	not enhanced	positive	N/A	N/A
Brooks et al.[9]	86	F	LA	N/A	slurred speech, swallowing difficulties, general weakness	high	low	hyperintense	not enhanced	negative	N/A	died
Obara [10]	72	M	LA	positive	dizziness, gait instability	high	low	hyperintense	not enhanced	negative	N/A	died
Yokota et al. [11]	60	F	LA	positive	headache, diplopia	high	low	hyperintense	not enhanced	positive	N/A	alive
Liu et al. [12]	68	F	LA	positive	vertigo, nausea, vomiting	high	normal	hyperintense	not enhanced	positive	N/A	died
Zoccarato et al. [13]	59	F	non-small cell lung cancer	N/A	headache, vomiting	high	low	hyperintense	not enhanced	positive	N/A	N/A
Huang et al. [14]	61	M	LA	positive	gait instability	high	low	hyperintense	not enhanced	positive	N/A	N/A
Tsukamoto et al. [15]	85	M	VZV meningoencephalitis	N/A	headache, fever, cognitive impairment	high	low	hyperintense	not enhanced	negative	positive	N/A
Tan et al.[6]	66	M	LA	positive	fever, behavioral change, confusion	high	low	hyperintense	not enhanced	positive	N/A	died
Chen et al. [16]	70	F	LA	positive	numbness, weakness in extremities	N/A	low	hyperintense	not enhanced	positive	N/A	alive
Tao et al.[17]	50	F	LA	positive	vertigo, nausea, vomiting	high	low	hyperintense	not enhanced	positive	N/A	N/A
Aubignat et al. [18]	62	F	LA	N/A	fever, disturbance of consciousness	high	N/A	hyperintense	not enhanced	positive	N/A	died
Haddar et al.[19]	46	M	LA	N/A	headache, vomiting	high	N/A	hyperintense	not enhanced	negative	N/A	died
Lenaerts et al. [20]	75	M	LA	BRAF V600E mutation	diplopia	high	low	hyperintense	not enhanced	positive	N/A	alive
Shaik et al. [21]	36	M	LA	positive	headache, blurring of vision, fever, loss of appetite, loss of weight	high	low	hyperintense	not enhanced	positive	N/A	alive
El Hasnaoui et al. [22]	46	M	LA	N/A	vomiting, dizziness, headache, weight loss	high	low	hyperintense	not enhanced	negative	N/A	died
Ray et al. [23]	48	M	LA	N/A	ataxia, facial nerve palsy and abducens nerve palsy	high	low	hyperintense	N/A	negative	negative	N/A
Present case	48	M	LA	positive	fever	high	low	hyperintense	not enhanced	negative	positive	died

*LA* lung adenocarcinoma, *DWI* diffusion-weighted imaging, *ADC* apparent diffusion coefficient, *T2WI* T2-weighted imaging, *FLAIR* fluid-attenuated inversion recovery, *Gd-T1WI* gadolinium-enhanced T1-weighted imaging, *CSF* cerebrospinal fluid, *VZV* varicella-zoster virus, *N/A* not available

VZV infection may involve the cranial nerves, most commonly the facial nerve, resulting in Ramsay Hunt syndrome, which is typically associated with facial palsy, sensorineural hearing loss, tinnitus, vertigo, nystagmus, and herpetic vesicular rash around the ear [25]. However, our patient had no clinical findings suggestive of facial nerve involvement, indicating that VZV meningitis rather than cranial neuropathy was the predominant neurological manifestation.

In our patient, the lack of contrast enhancement may support a mechanism involving perivascular or inflammatory changes rather than overt disruption of the blood–brain barrier, which is consistent with the vasculopathic features of VZV infection, particularly its involvement of small vessels [24]. Therefore, VZV infection may have contributed to the development of the bloomy rind sign through small-vessel involvement and perivascular inflammatory changes. However, the exact cause remains unknown, and further accumulation of cases is warranted. A limitation of the present report is that leptomeningeal dissemination was not pathologically confirmed because cerebrospinal fluid cytology remained negative on two separate examinations. Although the clinical presentation and MRI findings were highly suggestive of leptomeningeal dissemination, the diagnosis remained presumptive. Therefore, the relative contribution of leptomeningeal dissemination and VZV infection to the development of the bloomy rind sign could not be definitively determined. Furthermore, because this report describes a single case, the proposed association between VZV infection and the bloomy rind sign should be interpreted with caution. In addition, the literature review was based on a limited number of case reports and small case series and is therefore subject to publication bias and heterogeneous reporting.

## Conclusion

We report a case in which lung adenocarcinoma with VZV meningitis presented with the bloomy rind sign. On the basis of a review of previously reported cases, we highlight the characteristic imaging features of this finding and suggest that VZV infection may contribute to the development of the bloomy rind sign. However, this hypothesis is based on a single case and requires further validation.

## Data Availability

All relevant data are contained within the manuscript.

## Abbreviations

ADC: Apparent diffusion coefficient
CSF: Cerebrospinal fluid
DWI: Diffusion-weighted imaging
EGFR: Epidermal growth factor receptor
FLAIR: Fluid-attenuated inversion recovery
LA: Lung adenocarcinoma
MRI: Magnetic resonance imaging
PCR: Polymerase chain reaction
VZV: Varicella-zoster virus
